# Gaussian process emulation to improve efficiency of computationally intensive multidisease models: a practical tutorial with adaptable R code

**DOI:** 10.1186/s12874-024-02149-x

**Published:** 2024-01-27

**Authors:** Sharon Jepkorir Sawe, Richard Mugo, Marta Wilson-Barthes, Brianna Osetinsky, Stavroula A. Chrysanthopoulou, Faith Yego, Ann Mwangi, Omar Galárraga

**Affiliations:** 1 African Center of Excellence in Data Science, University of Rwanda, Kigali, Rwanda; 2 Academic Model Providing Access to Healthcare, Eldoret, Kenya; 3 Department of Epidemiology, Brown University School of Public Health, Providence, RI USA; 4 Department of Epidemiology and Public Health, Swiss Tropical and Public Health Institute, Basel, Switzerland; 5 Department of Biostatistics, Brown University School of Public Health, Providence, RI USA; 6 Department of Health Policy Management & Human Nutrition, Moi University School Public Health, Eldoret, Kenya; 7 Department of Mathematics, Physics & Computing, School of Science and Aerospace Studies, Moi University, Eldoret, Kenya; 8 Department of Health Services, Policy and Practice, and International Health Institute, Brown University School of Public Health, Providence, RI USA

**Keywords:** Tutorial, Emulation, Gaussian process, Bayesian analysis, HIV, Hypertension, Depression

## Abstract

**Background:**

The rapidly growing burden of non-communicable diseases (NCDs) among people living with HIV in sub-Saharan Africa (SSA) has expanded the number of multidisease models predicting future care needs and health system priorities. Usefulness of these models depends on their ability to replicate real-life data and be readily understood and applied by public health decision-makers; yet existing simulation models of HIV comorbidities are computationally expensive and require large numbers of parameters and long run times, which hinders their utility in resource-constrained settings.

**Methods:**

We present a novel, user-friendly emulator that can efficiently approximate complex simulators of long-term HIV and NCD outcomes in Africa. We describe how to implement the emulator via a tutorial based on publicly available data from Kenya. Emulator parameters relating to incidence and prevalence of HIV, hypertension and depression were derived from our own agent-based simulation model and other published literature. Gaussian processes were used to fit the emulator to simulator estimates, assuming presence of noise for design points. Bayesian posterior predictive checks and leave-one-out cross validation confirmed the emulator’s descriptive accuracy.

**Results:**

In this example, our emulator resulted in a 13-fold (95% Confidence Interval (CI): 8–22) improvement in computing time compared to that of more complex chronic disease simulation models. One emulator run took 3.00 seconds (95% CI: 1.65–5.28) on a 64-bit operating system laptop with 8.00 gigabytes (GB) of Random Access Memory (RAM), compared to > 11 hours for 1000 simulator runs on a high-performance computing cluster with 1500 GBs of RAM. Pareto k estimates were < 0.70 for all emulations, which demonstrates sufficient predictive accuracy of the emulator.

**Conclusions:**

The emulator presented in this tutorial offers a practical and flexible modelling tool that can help inform health policy-making in countries with a generalized HIV epidemic and growing NCD burden. Future emulator applications could be used to forecast the changing burden of HIV, hypertension and depression over an extended (> 10 year) period, estimate longer-term prevalence of other co-occurring conditions (e.g., postpartum depression among women living with HIV), and project the impact of nationally-prioritized interventions such as national health insurance schemes and differentiated care models.

**Supplementary Information:**

The online version contains supplementary material available at 10.1186/s12874-024-02149-x.

## Background

### The need for emulation

In situations where empirical data on disease impact(s) are not universally available or a randomized controlled trial may not be feasible, complex mathematical computer models (referred to as “simulators”) [1–3] can characterize disease prevalence, forecast incidence, and help identify cost-effective approaches for meeting short- and long-term health system needs [4–6]. Though simulation models play a critical role in helping public health decision-makers synthesize data from multiple sources and compare anticipated outcomes over time, the limitations of these models are not trivial. Simulation models based on central processing units (CPUs), such as grid computing and computing clusters, [7] require significant infrastructure that can incur high costs for hardware and oversight. Even with access to high-computing infrastructure, long run times of several hours for a single simulation and large numbers of input and output parameters can greatly inhibit fitting such models, [8] and in turn restrict analysts to considering only a subset of all possible simulated scenarios [9, 10]. Furthermore, because of their complexity, components of microsimulation models can still be perceived as a black box [11, 12] because their functions and behaviors are often not exhaustively described or immediately accessible at the time of publication, all of which makes it difficult for external users to interpret and adapt model processes for their local context.

Emulators are one tool that can help mitigate these limitations [9]. An emulator, also known as a metal-model, [13] is an approximation of one or more complex mathematical model(s) that is constructed using a training sample of simulator runs [14] and computationally more efficient. Emulators reduce costs by negating the need for super-computing infrastructure and, once developed, can substantially shorten the amount of time needed to implement model runs and interpret results.

In the last decade there has been an accelerated demand for integrated responses [15–20] to the growing burden of non-communicable diseases (NCDs) – including cardiovascular disease, cancers, diabetes, and mental illness – among people living with HIV (PLWH) in low and middle income countries (LMICs) [21–24]. In response to this call, the authors of this paper recently extended an established agent-based model of HIV transmission and treatment impact to include hypertension in two rural settings in Sub-Saharan Africa [24]. The authors’ simulation model was able to generate robust estimates of changing risks across age groups and predict growing population burdens of HIV and hypertension as comorbidities; however, the simulations were resource and time intensive and it is unlikely that novice modelers would be able to adapt the model’s components without input from an expert biostatistician. Other simulators of HIV and non-communicable diseases have met the same challenges [25]. To facilitate a greater understanding and usability of these complex models, we therefore share our experience developing an open-source emulator that approximates estimates from two simulators over an input subdomain of parameters related to HIV, hypertension and depression in a Sub-Saharan African (SSA) country with a generalized HIV epidemic. The tutorial presented in this paper (i) describes the steps involved in emulator development and validation, (ii) illustrates how to interpret the emulator’s predictive accuracy and outputs in relation to those from each simulator using case study data from Kenya, and (iii) provides annotated, open-source and adaptable R code to facilitate the use of the discussed methods in practice. We expect researchers with a basic understanding of Bayesian statistics and some familiarity with R software to be able to implement this protocol independently.

The emulation method described in this paper relies on well-established and validated Gaussian processes [14, 25, 26]. To the best of our knowledge, Gaussian-process emulation has not yet been used to mimic simulation models that predict the burden of HIV-comorbidities over time, nor has it been described in sufficient detail to enable use by non-biostatisticians. Thus, this tutorial is scientifically significant in that is uses a didactic approach to demystify Gaussian process emulation methods, and offers a new tool that can potentially improve public health decision-making with less resources.

## Methods: emulator development and validation

### Simulator description and source data

Evidence overwhelming indicates that the disproportionate burden of non-communicable diseases among people living with HIV – compared to individuals not living with HIV – will increase rapidly in the coming decades, [27, 28] and that most health systems in Sub-Saharan Africa are not currently equipped to treat the more than 15 million patients who require integrated HIV and NCD care [19]. In this tutorial we focus on two published simulation models that have estimated the future burden of HIV and non-communicable diseases in Kenya, as well as the costs and epidemiological impact of strengthening integrated care systems in the country. The two simulators were selected as examples for this tutorial because of their longer-term (i.e., > = 10 year) forecast periods and available details surrounding their design points. The two simulators were also selected because the rise of non-communicable diseases among persons living with HIV in Kenya is indicative and representative of the rise of NCDs on the continent, which are estimated to overtake infectious diseases worldwide by 2030 [19, 27, 28].

The first model is the authors’ Integrated Modeling of Epidemiologic and Economic Long-term Outcomes in Africa (inMODELA) microsimulation model, [29] which simulates HIV and hypertension in Kenya and South Africa from 2018 to 2028. The model is an extension of the Sexually Transmitted Diseases Simulator (STDSIM), [30, 31] a stochastic agent-based model that simulates transmission of HIV and other sexually transmitted diseases (STDs) through dynamic sexual networks. inMODELA was partly calibrated using population surveillance data of hypertension and HIV from western Kenya. National-level data on the hypertension prevalence were extracted from the 2015 Kenya STEPwise Approach to NCD Risk Factor Surveillance (STEPS) survey [32] while HIV modelling was calibrated using reports from 2007 and 2012 Kenya AIDS Indicator Surveys (KAIS) and the 2016 Kenya County HIV profiles, [33, 34] the most recent national data available at the time of simulator development. HIV was modelled as having four stages: early infection, asymptomatic, symptomatic and AIDS, and treatment with antiretroviral therapy was operationalized as individual ART demand and health system capacity to meet ART demand. Hypertension was modelled as being normotensive or hypertensive (i.e., blood pressure > 140/90 mmHg), accounting for the potential effects of age, gender, and economic development on hypertension risk. Key outputs of the inMODELA model include total annual mortality, incidence, and prevalence, as well as the health system burden of hypertension, HIV, and comorbid HIV and hypertension. Additional details of the inMODELA simulator are available in a separate publication [29].

The second model in this tutorial is an individual-based simulator initially developed by Smit et al. for Zimbabwe [35] and adapted for Kenya [36]. The model estimates current and future births, deaths, HIV disease and treatment, as well as prevalence/incidence of cardiovascular disease, chronic kidney disease, depression, diabetes, hypertension, and other NCDs and cancers among adults for the period of 2018 through 2035. Simulator calibration relied on data from the Joint United Nations Programme on HIV/AIDS, [37] 2016 Global Burden of Disease estimates [38] and other sources [36].

Relevant input parameters used to calibrate the two simulation models are summarized in Supplementary Table 1.

### Overview of the emulation process

**Fig. 1:**
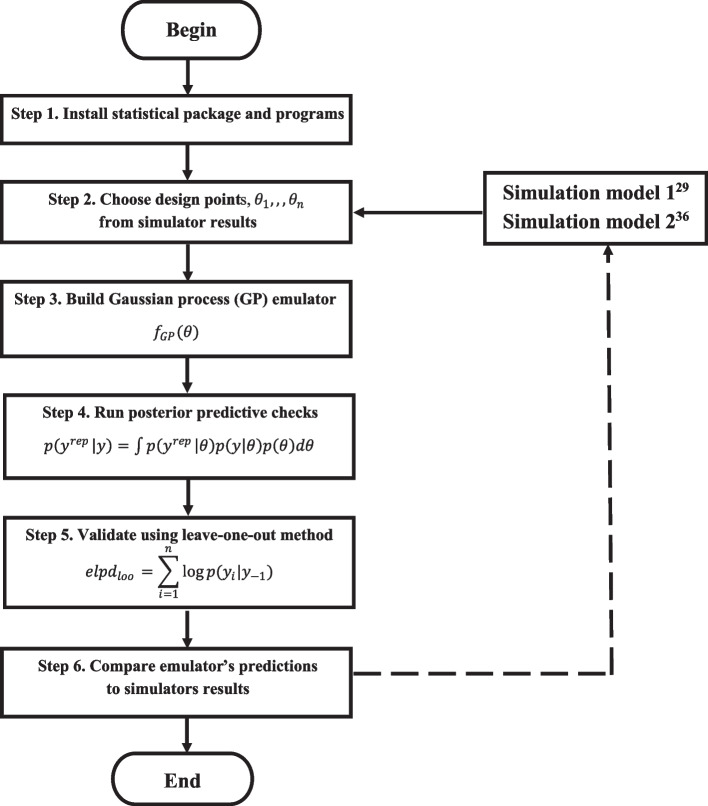
Overview of emulator development and validation process Figure 1 depicts the steps used to develop and validate the emulator approximating epidemiological HIV and NCD data from two established simulators. First, the R statistical package is installed. Second, relevant parameters related to the 10-year prevalence of HIV, hypertension, comorbid HIV and hypertension, and depression are abstracted from published simulators to serve as the emulator’s design points, and entered into R. Third, Gaussian processes (GP) approximate the mean and variance of each simulator’s outputs, assuming presence of noise for the emulator’s design points. Fourth, Bayesian posterior predictive analysis is used to infer the credibility of future HIV and NCD prevalence(s) based on the posterior distribution. Fifth, leave-one-out cross validation confirmed the emulator’s predictive accuracy. Lastly, the emulator’s predictions are compared and interpreted in relation to those of the simulator(s)

Figure 1 summarizes the steps that were used to develop and validate an emulator to approximate epidemiological outputs projected over 10 years by the multidisease simulators. To develop the emulator, we first abstract relevant parameters from the more complex simulation models to serve as the emulator’s design points. In this example, parameters related to the prevalence of HIV, hypertension, comorbid HIV and hypertension, and depression among PLWH were selected. Prevalence parameters were ascertained for 2018 and for 2028. Second, we use Gaussian processes (GP) to approximate the mean and variance of each simulator’s outputs, assuming presence of noise for our design points. Third, we use Bayesian posterior predictive analysis to analyse the credibility of future emulator predictions based on the posterior distribution. Lastly, we use leave-one-out cross validation to confirm the emulator’s predictive accuracy and compare emulator estimates to simulation results.

### Step 1. Install the program

This tutorial uses the GauPro package [39], the rstanarm package [40] and the loo package [41, 42] in R version 4.1.2 for emulator construction and application. To replicate results from this tutorial, or to adapt this emulator to new simulation data, the latest version of the free R software environment needs to be installed, and can be downloaded from https://www.r-project.org/.

### Step 2. Select key design points from simulation model(s)

Not all parameters of a complex simulation model will be needed for emulation. Only those most informative for your research question should be ascertained. In this example using simulator data from Kenya, parameters relating to the prevalence of HIV, hypertension, and comorbid HIV and hypertension were ascertained from the inMODELA simulation model [29] for the period of 2018 through 2028. Parameters relating to the prevalence of depression among people living with HIV were ascertained from the Smit et al. model [36] for the period of 2018 through 2030. (Table 1) To reconcile the different forecasting periods used by the two simulation models, we assumed the prevalence of depression in 2028 to be the average prevalence for years 2025 and 2030. Supplementary Table 2 specifies the design points used to emulate annual HIV and NCD prevalence for the period of 2018 through 2028.

**Table 1:** Summary of final design points used for emulation, based on example national data from Kenya

Simulator Output Description	Simulation year		
	2018	2028	Source
Annual incidence of hypertension	3.90% (3.8–4.1)	4.20% (4.0–4.3)	[29]
Annual incidence of HIV	0.85% (0.83–0.87)	0.37% (0.36–0.38)	[29]
Annual incidence of HIV or hypertension among individuals who already have the other disease (comorbid HIV and hypertension)	0.37% (0.33–0.39)	0.37% (0.33–0.39)	[29]
Prevalence of hypertension	29.47% (0.28–0.31)	34.30% (0.33–0.35)	[29]
Prevalence of HIV	4.81% (0.04–0.05)	2.55% (0.02–0.03)	[29]
Prevalence of comorbid HIV and hypertension	2.06% (0.02–0.03)	1.31% (0.01–0.02)	[29]
All-cause mortality	~ 1500 per 100,000	~ 1500 per 100,000	[29]
Depression prevalence among people living with HIV	3.90%	3.70%^a^	[36]

^a^ Prevalence of depression in 2028 represents the average prevalence between years 2025 and 2030. Numbers in parenthesis represent 95% uncertainty ranges

Update the emulator’s R code to reflect relevant design points and corresponding emulation time period(s) abstracted from simulator data.

### Step 3. Fit Gaussian processes to simulation data

A Gaussian process refers to a collection of any finite number of random variables which have a Gaussian (normal) distribution [25]. A GP emulator uses a statistical model to fit a Gaussian process to a dataset, and is defined by (1) a mean function describing the mean at any point of the input space and (2) a covariance function describing the covariance between points [25, 39]. When emulating a stochastic simulator, the unknown function is assumed to be the expectation of the *i*^*th*^ simulator output denoted as *f*_*i*_(*x*).

We construct our GP emulator such that, for each simulator output *f*_*i*_(*x*), we select active variables (*x*^*A*^) and then emulate using the following process:

1 $${f}_i(x)=\sum\nolimits_{i=1}^q{\beta}_i{g}_i\left({x}^A\right)+{\mu}_i\left({x}^A\right)+{\delta}_i(x).$$

The first part of the emulator, $\sum_{i=1}^q{\beta}_i{g}_i\left({x}^A\right),$ is a polynomial of active inputs *x*^*A*^ running from *i* = 1, …*q* chosen from the simulator outputs, *β*_*i*_ are the regression coefficients, and *g*_*i*_(˙) are the deterministic functions of *x*^*A*^ which are known [43]. The second part of the emulator, *μ*_*i*_(*x*^*A*^), represents a collection of any finite number of random variables which have a Gaussian distribution [43]. Supposing that *μ*_*i*_(*x*^*A*^) is a Gaussian process with zero mean and known variance, we can define it as:

2 $$\mu \left({x}^A\right)\sim GP\left(0,c\left({x}^A,{x^A}^{\prime}\right)\right)$$

where *c*(*x*^*A*^, *x*^*A*′^) is a covariance function that determines the relationship between *μ*(*x*^*A*^) and *μ*(*x*^*A*^)^′^ based on the distance between *x*^*A*^ and *x*^*A*′^. The Gaussian process used to develop the current emulator has a covariance structure given by:

3 $$Cov\left({\mu}_i\Big({x}_1^A\right),{\mu}_i\left({x}_2^A\right)\Big)={\sigma}_i^2\exp \left[\frac{-{\left|{x}_1^A-{x}_2^A\right|}^2}{\theta_i^2}\right].$$

The parameter *σ* in eq. 3 can be varied to obtain the desired amount of waves in the emulator, whereby a smaller value of *σ* results in less extreme waves [44]. *θ* > 0 are unknown correlation length parameters where large values of *θ* indicate a smooth output function of the *i*^*th*^ input and small values suggest high non-linearity [45]. The last part of the emulator, *δ*_*i*_(*x*), models the effects of inactive variables as random noise.

For each output of interest, the emulator provides the expectation E [*f*_*i*_(*x*)] and variance *var*(*f*_*i*_(*x*)) at *x* for every output given by *i* = 1, 2, …*n* where *x* denotes a vector of the emulator inputs. We evaluate *f*_*i*_(*x*) as the prevalence value(s) projected from 2018 to 2028 for HIV, hypertension, comorbid HIV and hypertension, and depression. The results are gathered into a vector *D*: ${D}_i=\Big(f{\left({x}_1^A,\dots, f\left({x}_q^A\right)\right)}^T$ where *q* represents the number of design points. The emulator is then plotted as an adjusted expectation and variance function of *f*_*j*_(*x*): *E*_*D*_(*f*_*j*_(*x*)) and *Var*_*D*_(*f*_*j*_(*x*)).

### Step 4. Run graphical posterior predictive checks of emulator fit

In this step, we use graphical displays to check that the disease burden trends produced by our emulator look similar to the simulator data we observed. Our emulator uses Bayesian posterior predictive analysis to assess the credibility of future observable data based on the posterior distribution [46]. We analyze the Bayesian posterior distributions graphically to check predictive accuracy of the posterior distributions, plotting simulated *y* values from the posterior distribution against the actual values of *y*. The posterior predictive distribution is defined as:

4 $$p\left({y}^{rep}|y\right)=\int p\left({y}^{rep}|\theta \right)p\left(y|\theta \right)p\left(\theta \right) d\theta$$

where *y*^*rep*^ represents future data that could be drawn from the posterior distribution, *y* is the current simulator data, *θ* is the model parameter, and *p*(*y*| *θ*) is the sampling density for future data conditional on the parameter [46]. The size of the posterior sample was based on 50 draws which showed to be sufficient enough to generate the *y*^*rep*^ matrix from the posterior predictive distribution [40].

### Step 5. Validate the emulator’s accuracy

We use the Bayesian leave-one-out cross validation (LOOCV) technique to confirm the emulator’s predictive accuracy. The LOOCV method applies the log-likelihood evaluation of posterior parameter values, [42] and is appropriate for smaller data sets and when similar distributions exist in the training and testing data [47]. LOOCV assesses the predictive ability of posterior simulations in which the data is iteratively partitioned into either calibration (training) sets or validation (test) sets. LOOCV is one of the most accurate ways to estimate how well a model will perform on unseen, “out-of-sample” data [42]. The calibration set is used to train the model and produce output values that are compared with the test set [48]. We validated the emulator’s accuracy using the loo package in R whereby the computation uses Pareto-smoothed importance sampling (PSIS) to regularize importance weights [42]. Following prior work [42, 49], a Pareto *k* estimate less than or equal to a 0.70 threshold was used to confirm reliability of the emulator’s performance. The LOOCV approach is specified in eqs. 5–7.

Given that data *y*_1_, …*y*_*n*_ are independently modelled given the parameters θ then, $p\left(y|\theta \right)={\prod}_i^np\left({y}_i|\theta \right)$. Suppose a prior distribution *p*(*θ*) gives a posterior distribution *p*(*θ*| *y*) and a posterior predictive distribution *p*(*y*˜| y) = ∫ *p*(*y*˜_*i*_|*θ*)*p*(*θ*| *y*)*dθ*. A predictive accuracy measure for *n* data points termed as expected log pointwise predictive density (elpd) for a new dataset is given as:

5 $$\sum\nolimits_1^n\int {p}_t\left(y{\sim}_i\right)\mathit{\log}\ p\left(y{\sim}_i|y\right) dy{\sim}_i$$

where *p*_*t*_(*y*˜_*i*_) is the distribution of the real data generation for *y*˜_*i*_. The values of *p*_*t*_(*y*˜_*i*_) are unknown and therefore cross-validation is used to approximate (5). The Bayesian leave-one-out (LOO) estimate of the predictive fit is:

6 $${elpd}_{loo}=\sum\nolimits_{i=1}^n\log p\left({y}_i|{y}_{-1}\right)$$

where

7 $$p\left({y}_i|{y}_{-1}\right)=\int p\left({y}_i|\theta \right)p\left(\theta |{y}_{-1}\right) d\theta$$

is the leave-one-out predictive density given the data without the *i*^*th*^ data point.

To train the emulator, we followed the Emulation and History Matching Handbook for R [50] whereby we chose a number of training points equal to ten times the number of parameters. Given that we were estimating only one parameter (prevalence) for each of the four outcomes of interest, this resulted in 10 training points.

## Results: emulator interpretation

Using outputs from the two simulation models of HIV, hypertension and depression burden, our emulator shows to be as accurate and computationally more efficient at predicting prevalence of these comorbidities in Kenyan populations over 10-years. On average, the inMODELA simulator took 11 hours and 53 minutes to perform 1000 runs on a high-performance computing cluster (HPC) and used 208 central processing units (CPU) cores, 2 graphics processing units (GPUs), and 1500 gigabytes (GBs) of random-access memory (RAM). The model by Smit et al. was coded in C++, ran using Xcode (Mac), and took a little over 3 minutes per iteration on an 8-core machine. Although run times were not overwhelmingly long, the Smit et al. simulation model faced computational constraints due to the large size of model outputs; this necessitated an additional post-processing step of aggregating and summarizing each batch of 10 model iterations in MatLab. By comparison, the present emulator was developed and validated on a Hewlett-Packard Intel Core i5 laptop with a 64-bit operating system, 2.50 CPU of 2.50 GHz and 8.00 GBs of RAM, and took only a few seconds for a single run using the same statistical program for development, validation and output interpretation.

**Fig. 2:**
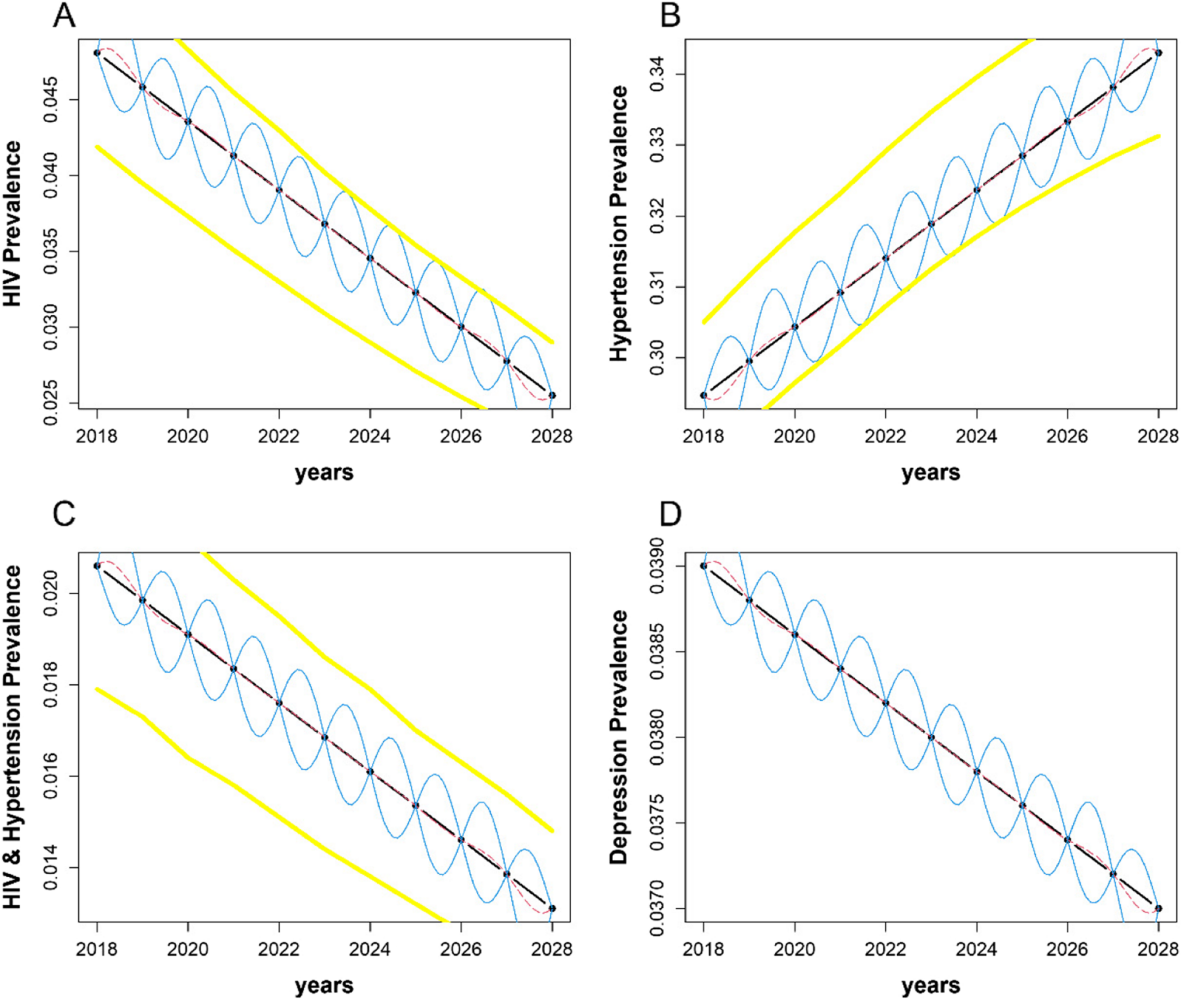
Gaussian process emulator for (**a**) HIV, (**b**) Hypertension, (**c**) Comorbid HIV & Hypertension and (**d**) Depression in Kenya At each year, black dots represent the selected design points used to fit the emulator, the blue curved lines represent the predicted 95% uncertainty intervals for each design point, the dashed red lines are the mean prevalence for a given year plotted as a function presenting the emulator’s predictions and the yellow thick lines outside are the 95% confidence intervals for the original simulators. Sub-plot D does not include the 95% confidence intervals for the simulator because these data were unpublished

Figure 2 shows the emulator’s projected mean prevalence over time for the example data in terms of: (A) HIV, (B) hypertension, (C) comorbid HIV & hypertension, and (D) depression among adults living with HIV. In each sub-figure of Fig. 2, black points should be interpreted as the design points (i.e., simulator outputs) used to fit the emulator, dashed red lines show the mean prevalence plotted as a function of the emulator’s predictions, solid blue lines represent the 95% Confidence Intervals (CI) for the emulator’s mean estimates, and the yellow lines are the 95% Confidence Intervals for the simulators’ mean estimates (Confidence Intervals for the annual mean prevalence of depression among PLWH were not publically available). We can then visually understand the emulated trends in disease burden to closely reproduce the trends projected by each simulator. For example, the emulator predicts that the mean prevalence of HIV will be 0.02776 [95% CI: 0.02774–0.02778] in the year 2027 compared to 0.02750 [95% CI: 0.02379–0.03121] predicted by the inMODELA simulator. The pointwise confidence intervals used for statistical uncertainty quantification are wider between each emulation year than at each annual point estimate (e.g., at the midpoint between 2027 and 2028, the mean prevalence of HIV is predicted to be 0.02573 (95% CI: 0.02219, 0.02928). The width of the credibility interval is largest between points and approaches zero near each point estimate due to the Gaussian process regression towards the mean. When considering the prevalence(s) of HIV, hypertension, and comorbid HIV & hypertension in this example, the emulator’s 95% Confidence Intervals for each mean estimate are very similar to those projected by the inMODELA simulator. Given the linearity and lack of noise in the original data, the emulator’s uncertainty ranges are within those of the inMODELA estimates. This indicates that our emulator accurately approximates outputs from the more complex model.

**Fig. 3:**
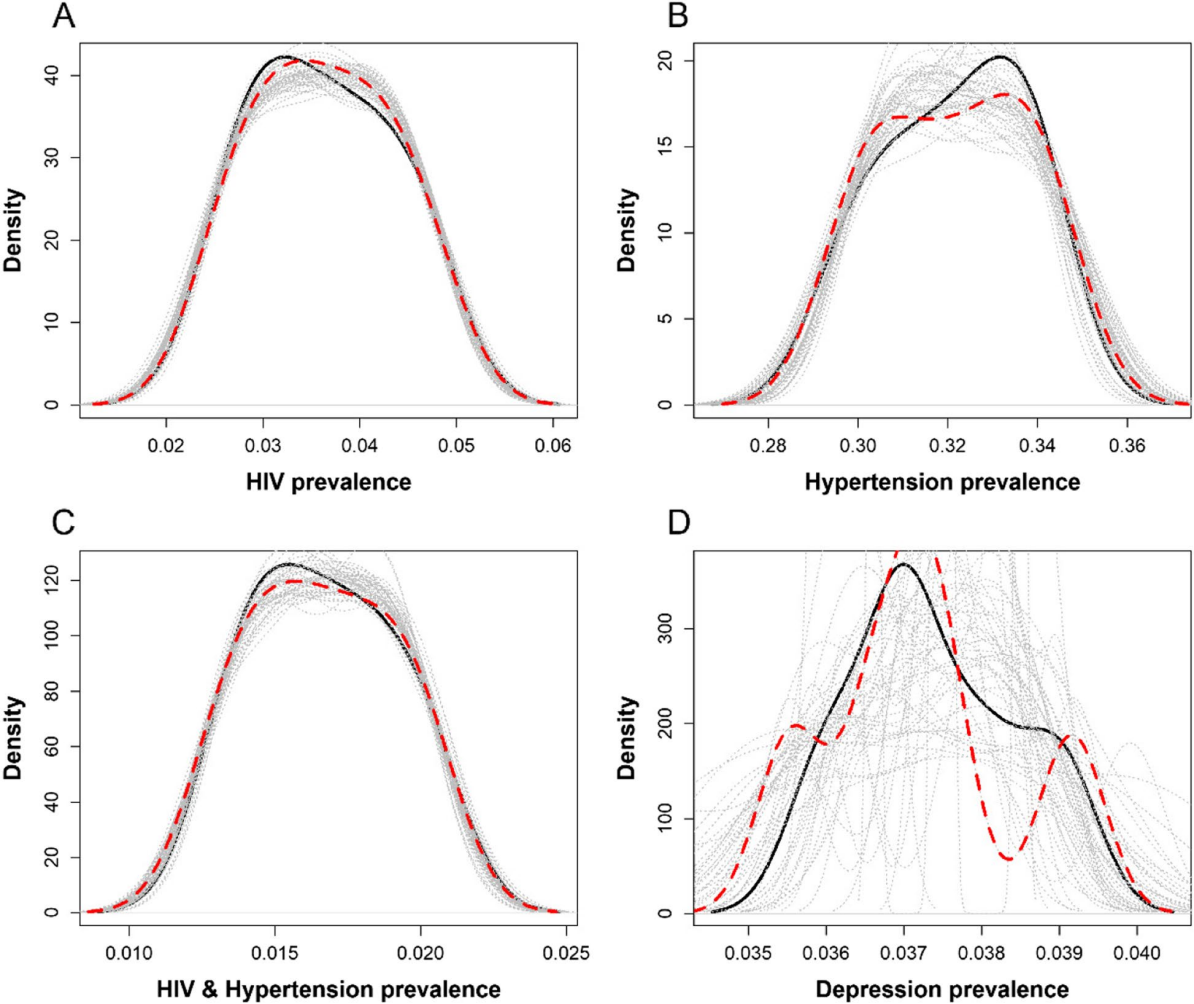
Gaussian posterior predictive distributions for (**a**) HIV, (**b**) Hypertension, (**c**) Comorbid HIV & Hypertension and (**d**) Depression prevalence The solid black lines represent the distribution of y ; the dashed red lines represent the y posterior predictive distribution and the dotted grey lines represent the distribution of simulations. The density values of the plots are different as generated by the system due to the differences in disease-specific prevalence values on the x-axis

Figure 3 shows the Gaussian posterior predictive distributions for the example Kenyan data for HIV, hypertension, comorbid HIV & hypertension, and depression. The solid black lines can be understood as the distribution of *y*; the dashed red lines represent the *y* posterior predictive distribution, and the dotted grey lines represent the distribution of the simulations performed. We observe from panel A, B, C and D in Fig. 3 that the posterior predictive distributions denoted by *post-pred y* are not far off from the current fitted data denoted by *y*. Density values differ for each panel due to different prevalence values for the respective disease(s) on the x-axis, with greater imprecision for depression estimates (panel D) because of fewer available simulation data at each projection year.

For each output of interest, the LOOCV yielded Pareto *k* diagnostic values of < 0.70, indicating practical convergence rates and reliable Monte Carlo error estimates. Validating diagnostics for each of the emulator’s output are provided in Supplementary Figures 1–4.

Supplementary Figure 5a shows the emulator’s projected mean prevalence of HIV when the simulation model assumes that Kenya’s ART coverage targets are achieved (i.e., 90% of people with HIV are aware of their status, and, of those, 90% are enrolled in HIV care^1^) due to the implementation of effective interventions along the care continuum. Supplementary Figure 5b shows the emulator’s projected mean prevalence of hypertension when Kenya’s hypertension treatment targets are reached (i.e., when 50% of persons with confirmed hypertension are enrolled in care and receiving drug therapy and counselling). Supplementary Figures 5c and 5d show the projected disease prevalences when one or both of these treatment targets are achieved. Supplementary Figure 6 shows the Gaussian posterior predictive distributions for these emulations. When considering the prevalence(s) of HIV and hypertension in the presence of hypothetical interventions that help achieve treatment targets, the emulator’s 95% confidence intervals for each mean estimate closely approximate those projected by the inMODELA model. Similar to emulator outputs based on disease prevalence simulations in the absence treatment, the emulator’s uncertainty ranges for prevalence estimates in the presence of wider treatment uptake are within those of the inMODELA simulation estimates. This indicates that our emulator accurately approximates outputs from more complex modelling of the disease-impact of chronic disease intervention(s). We also see a substantial lack of density values for each panel for the respective disease(s) on the x-axis in Supplementary Figure 6, further indicating high precision of the emulator for estimating treatment impacts on chronic disease burden over time. For all emulations of disease burden in the presence of higher intervention uptake, the Pareto k diagnostic values were < 0.70 (Supplementary Figures 7–10).

## Discussion

### Observed accuracy and efficiency

Applying widely-used Gaussian processes, this tutorial details the steps used to develop a new, user-friendly emulator that can approximate multi-year estimates of chronic disease burden from two computationally intensive simulation models. We demonstrate that the emulator closely reproduced trends in disease burden projected by the published simulators from which our parameters were sourced [29, 36]. In this example, from 2018 through 2028, prevalence of HIV and depression among PLWH in Kenya is projected to decrease by approximately 2 and < 1 percentage points, respectively, with prevalence of hypertension increasing by 5 percentage points over the same 10-year forecast. Successful validation checks (Pareto *k* estimates < 0.70 for all emulations) confirmed the emulator’s predictive accuracy. Disease prevalence estimates were modelled in only a few seconds on a 64-bit operating system laptop with 2 CPU cores of 8 GBs of RAM using the emulator presented in this paper, while simulations [29] took more than 11 hours to perform 1000 runs on a high-performance computing cluster with 208 CPU cores, 2 GPUs and 1500 GBs of RAM. Thus, our emulator was able to more efficiently model disease burden over time without compromising the statistical accuracy of more computationally intensive simulators. Outputs from sensitivity analyses suggest that the emulator is equally efficient and reliable for approximating simulations of disease burden in the presence of effective treatment interventions.

### Future applications

The emulator presented in this paper was developed and validated using 10-year demographic and epidemiological case study data from Kenya. However, the broader Gaussian processes described in this tutorial (and made available via the open source R code) are widely validated in the fields of public and environmental health as reliable methods for emulating results of complex and resource-intensive models, [9, 43, 49] including for those above and beyond Kenyan populations and adults living with chronic conditions. For example, a tutorial using HIV data from Uganda [8] found that history matching and emulation of an 18 output simulator had a 65% probability of fitting all simulator outputs and was several orders of magnitude faster to evaluate. A Bayesian optimization emulator with Gaussian processes [51] has similarly shown to adequately capture the input–output relationship of the *OpenMalaria* individual-based model (IBM) [52] of malaria transmission while improving upon the simulator’s overall goodness of fit. And a Gaussian process emulator of an IBM of microbial communities [53] has demonstrated an approximately 220-fold increase in computational efficiency, with the percentage of variance explained by the univariate emulator ranging from 83 to 99%. Thus, through the selection of alternative design points, the emulator in this tutorial has the potential to approximate other simulators outside of those for HIV, hypertension and diabetes. As is the goal with any emulation exercise, our emulator offers a statistical model that can be used as a surrogate for chronic disease simulators. Because our simple emulator showed to be valid and more efficient in mimicking 10-year prevalence of HIV comorbidities in the absence of intervention, the next step is to evaluate how the emulator will also be able to mimic future simulators that model disease burden in the presence of targeted interventions. For example, Hamilton et al. developed an agent-based simulation of HIV-1 transmission in Kenya to estimate the potential population-level impact of providing PrEP layered into standard care services over 10 years [54, 55]. These and other longitudinal simulator data offer ideal design points to further test the emulator’s predictive accuracy for modelling intervention effects on HIV treatment and prevention. Several microsimulation models have been developed to characterize and plan for the rapidly growing burden of non-communicable diseases in SSA [56–58] and elsewhere [59–61]. Also, though still in early stages, recent advances in computing power are now allowing large and complex ABMs to be simulated in reasonable amounts of time using desktop GPUs [62]. Yet, to the best of our knowledge, none of these NCD models have been made available to local public health decision makers through accessible and understandable platforms, which the present emulator may begin to correct.

### Limitations and strengths of this tutorial

There are some limitations to the emulation methods presented in this paper. First, Gaussian process emulation was appropriate given the small number of design points being considered in this tutorial. However, GP emulators do not scale well when including many (e.g., > 50) inputs [9, 63, 64], such that several lower-dimensional emulators might be more appropriate when a greater number of simulator outputs are being considered [9]. Relatedly, because our original simulation model data were essential linear, the emulator increased the efficiency of our predictions (i.e., narrower uncertainty ranges). It is possible that our emulator may lose computational efficiency or yield estimates with greater uncertainty when applied to higher dimensional data or to data with asymmetric noise. Incorporating interval calibration [65], Monte Carlo [66], and other methods in future iterations of the emulator could help address this limitation. Second, we fit separate univariate GPs emulators to model each simulator output individually, which neglects any potential correlation [67] between the outputs [53] (e.g., between changes in the prevalence of hypertension and depression among PLWH over time). Future expansions of the emulator can address this issue by using a multivariate Gaussian process [68]. Lastly, as is common to mathematical modelling work, the simulation models selected for this tutorial suffered from incomplete surveillance data which restricted our ability to perform additional emulation procedures such as history matching [69] to reduce simulator input space or additional diagnostic checks [70] that rely on more robust training data.

Despite these limitations, the emulator presented in this paper offers an accessible tool for health policy makers who may not have a background in disease modelling. This will help to build the modelling capacity of local decision makers who are working to build integrated HIV and chronic disease programmes with limited resources [71]. While transparency surrounding microsimulation model development has increased in recent years as these models become more widespread, it is often impractical to document every detail of their functionality [11, 12]. Emulators do not address all transparency concerns related to black box modelling, but they can help address concerns related to communicating results from complex models for wider audiences. Thus, a key strength of this emulator is its simplicity; the step-by-step annotated code that was programmed using free R software and is available via an open-source repository can encourage future use and adaption at no cost to the user.

Secondly, our emulator uses Bayesian inferences rather than a frequentist approach in the posterior predictive analysis, which maximizes both the prior and currently available data. Third, we used the leave-one-out cross validation technique which offers an unbiased and reliable diagnostic check when similar distributions exist in the training and test data [47, 49, 70]. Fourth, our emulator fills a gap in the health decision-making toolbox in that it is one of the first to model the dual burden of HIV and hypertension and of HIV and depression for a country with a generalized HIV epidemic and growing non-communicable disease burden.

## Conclusion

This emulation tutorial responds to calls from international donors and global health researchers [72] to “*make modelling tools and analytic packages publicly available to wider audiences*” and facilitate “*training of decision makers to understand model outputs, particularly uncertainty and confidence intervals*”. We envision future expansions of this emulator to be able to estimate changes in HIV and NCD burden with greater coverage of National Health Insurance schemes and in the presence of integrated care interventions [73, 74], and to estimate the cost-effectiveness of integrated interventions based on current [6, 75] and emerging data.

## Data Availability

All annotated code used to develop and run the emulator presented in this paper is freely available via the following digital repository: 10.7910/DVN/LUBYHQ

## Supplementary Information

**Additional file 1.**

## Abbreviations

CPU: Central Processing Unit
GB: Gigabyte
GP: Gaussian Process
GPU: Graphics Processing Unit
HIV: Human Immunodeficiency Virus
inMODELA: Integrated Modeling of Epidemiologic and Economic Long-term Outcomes in Africa
KAIS: Kenya AIDS Indicator Surveys (KAIS)
LMICs: Low- and Middle-Income Countries
LOOCV: Leave-One-Out Cross Validation
NCD: Non-communicable Disease
PLWH: People Living with HIV
RAM: Random Access Memory
SSA: Sub-Saharan Africa
STD: Sexually Transmitted Disease
STDSIM: Sexually Transmitted Diseases Simulator

## Footnotes

1 The simulation model was developed and published in 2019, at which time the UNAIDS 90–90-90 targets were in effect in Kenya.
