# Supplementary material for: Gaussian process emulation to improve efficiency of computationally intensive multidisease models: a practical tutorial with adaptable R code
Source: BMC Med Res Methodol. 2024 Jan 27;24:26. doi: 10.1186/s12874-024-02149-x (PMC10821551; doi:10.1186/s12874-024-02149-x)

**Supplementary Information Files for the manuscript:**

**Gaussian process emulation to improve efficiency of computationally intensive multidisease models: A practical tutorial with adaptable R code**

**Authors:** Sharon Jepkorir Sawe^1^, Richard Mugo^2^, Marta Wilson-Barthes^3^, Brianna Osetinsky^4^, Stavroula A. Chrysanthopoulou^5^, Faith Yego^6^, Ann Mwangi^2,7^, Omar Galárraga^2,8§^

^1^ African Center of Excellence in Data Science, University of Rwanda, KK 737 St. Kigali, Rwanda; [sharonsawe21@gmail.com](mailto:sharonsawe21@gmail.com)

^2^ Academic Model Providing Access to Healthcare, P.O. Box 4606 Eldoret, Kenya 30100; [rngarigo@gmail.com](mailto:rngarigo@gmail.com)

^3^ Department of Epidemiology, Brown University School of Public Health, 121 S. Main St. Providence, Rhode Island 02912, United States; [marta_wilson-barthes@brown.edu](mailto:marta_wilson-barthes@brown.edu)

^4^ Department of Epidemiology and Public Health, Swiss Tropical and Public Health Institute, Kreuzstrasse 2, 4123 Allschwil Basel, Switzerland; [brianna.osetinsky@swisstph.ch](mailto:brianna.osetinsky@swisstph.ch)

^5^ Department of Biostatistics, Brown University School of Public Health, 121 S. Main St. Providence, Rhode Island 02912, United States; [stavroula_chrysanthopoulou@brown.edu](mailto:stavroula_chrysanthopoulou@brown.edu)

^6^ Department of Health Policy Management & Human Nutrition, Moi University School Public Health, P.O. Box 4606 - 30100, Eldoret, Kenya; [yegofaith@gmail.com](mailto:yegofaith@gmail.com)

^7^ Department of Mathematics, Physics & Computing, School of Science and Aerospace Studies, Moi University, P.O. Box 3900-30100, Eldoret, Kenya; [annwsum@gmail.com](mailto:annwsum@gmail.com)

^8^ Department of Health Services, Policy and Practice, and International Health Institute, Brown University School of Public Health, 121 S. Main St. Providence, Rhode Island 02912, United States; [omar_galarraga@brown.edu](mailto:omar_galarraga@brown.edu)

^§^ **Corresponding Author:**

Omar Galárraga, PhD

Associate Professor of Health Services, Policy & Practice

Brown University School of Public Health

121 South Main Street, Box G-S121-2

Providence, RI 02912

[omar_galarraga@brown.edu](mailto:omar_galarraga@brown.edu)

**Supplementary Table 1. Summary of key parameters used to calibrate microsimulation models of HIV, hypertension and depression prevalence and incidence in Kenya**

| Simulator Input Component | Input Description | Source/value/distribution | Basis for choice | Source |
| --- | --- | --- | --- | --- |

| **Demography** | Life Table | Global Burden of Disease 2016 age-sex specific mortality | Able to estimate mortality in absence of hypertension and HIV | ^26^ |
| --- | --- | --- | --- | --- |
|  | Fertility Table | Kenya Demographics and Health Surveys | Best available estimate | ^26^ |
| **Sexual Behavior** | Age group sexual behavior | Iterated promiscuity multiplier for male and female individual age groups to reflect sexual behavior differences by age and sex | Adapted to fit age specific trends in HIV prevalence imputed from combination of KAIS and Kenya County Report | ^26^ |
|  | Promiscuity factor (sexual behavior multiplier) | 0∙9  Iterated from 0∙5 to 1∙2 to find best fit | Calibrated to fit age specific trends in HIV prevalence | ^26^ |
| **Natural History of Disease** | Hypertension Baseline  Male | Weibull Distribution Parameterized as  *t_0_ = 1∙8(x-64)e(-(64)^1∙8^)* | Fitting. Sensitivity parameters varied  µ = age, 62-66  β = shape, 1∙7-1∙9 | ^26^ |
|  | Hypertension Baseline  Female | Weibull Distribution Parameterized as  *t_0_ = 2∙2(x-55)e(-(63)^2∙2^)* | Fitting. Sensitivity parameters varied  µ = age, 53-57  β = shape, 2∙1-2∙3 | ^26^ |
|  | Hypertension Modernization Male | Weibull Distribution Parameterized as  *t_0_ = 1∙5(x-40)e(-(40)^1∙5^)* | Fitting. Sensitivity parameters varied  µ = age, 38-42  β = shape, 1∙3-1∙7 | ^26^ |
|  | Hypertension Modernization Female | Weibull Distribution Parameterized as  *t_0_ = 1∙5(x-50)e(-(50)^1∙5^)* | Fitting. Sensitivity parameters varied  µ = age, 48-52  β = shape, 1∙3-1∙7 | ^26^ |
|  | Hypertension Menopausal Bump | Weibull Distribution Parameterized as  *t_a_ = 2(x-57)e(-(57)^2^)*  offset = 37 | Fitting. Sensitivity parameters varied  µ = age, 55-59  β = shape, 1∙8-2∙2  offset = 35-39 | ^26^ |
|  | Hypertension Mortality Male | Weibull Distribution Parameterized as  *t_a_ = 2(x-63)e(-(63)^2^)* | Framingham Risk Score 10 Year survival.  Age = 45  BMI = 23∙4  SBP = 148  Smoking = No  Diabetic = No | ^26^ |
|  | Hypertension Mortality Female | Weibull Distribution Parameterized as  *t_a_ = 2(x-63)e(-(63)^2^)* | Framingham Risk Score 10 Year survival.  Age = 46  BMI = 25  SBP = 151  Smoking = No  Diabetic = No | ^26^ |
|  | Modernization/  Menopause  multiplier | 0∙1  Sensitivity analysis 0∙0-0∙12 | Iterated from 0 to 1 Best fit | ^26^ |
| **Treatment** | Coverage = access * linkage | 0∙77 | Coverage percent of HIV+ linked to care in western Kenya, Kenya Strategic report | ^26^ |
|  | Depression Crude prevalence (95% CI) | 8·5% (5·1% - 11·8%) |  | ^33^ |

**Supplementary Table 2. Final design points used for emulator development and validation, by simulation year**

| Simulator Output Description | Simulation year | | | | | | | | | | | | | |  | |
| --- | --- | --- | --- | --- | --- | --- | --- | --- | --- | --- | --- | --- | --- | --- | --- | --- |
|  | 2018 | 2019 | 2020 | 2021 | 2022 | 2023 | 2024 | 2025 | 2026 | 2027 | 2028 | 2030 | 2035 | Source | |  |
| Prevalence of hypertension | 29.47%  (0.285-0.305) | 30.12%  (0.291-0.312) | 30.71%  (0.297-0.318) | 31.25%  (0.302-0.323) | 31.82%  (0.307-0.329) | 32.36%  (0.313-0.335) | 32.83%  (0.317-0.340) | 32.27%  (0.321-0.344) | 33.65%  (0.325-0.348) | 34.00%  (0.328-0.352) | 34.30%  (0.331-0.355) | - | - | ^26^ | |  |
| Prevalence of HIV | 4.81%  (0.042-0.054) | 4.54%  (0.040-0.051) | 4.28%  (0.037-0.048) | 4.03%  (0.035-0.045) | 3.80%  (0.033-0.043) | 3.55%  (0.031-0.040) | 3.34%  (0.029-0.038) | 3.13%  (0.027-0.035) | 2.94%  (0.025-0.033) | 2.75%  (0.024-0.031) | 2.55%  (0.022-0.029) | - | - | ^26^ | |  |
| Prevalence of comorbid HIV and hypertension | 2.06%  (0.018-0.023) | 1.98%  (0.017-0.022) | 1.88%  (0.016-0.021) | 1.81%  (0.016-0.020) | 1.73%  (0.015-0.020) | 1.65%  (0.014-0.019) | 1.58%  (0.014-0.018) | 1.51%  (0.013-0.017) | 1.44%  (0.013-0.016) | 1.38%  (0.012-0.016) | 1.31%  (0.011-0.015) | - | - | ^26^ | |  |
| Depression prevalence among people living with HIV | 3.90% | - | 3.8% | - | - | - | - | 3.7% | - | - | **-** | 3.7% | 3.8% | ^33^ | |  |

95% credible intervals in parentheses.

**Supplementary Figure 1. Diagnostics of emulator’s predictive accuracy for modeling HIV prevalence**


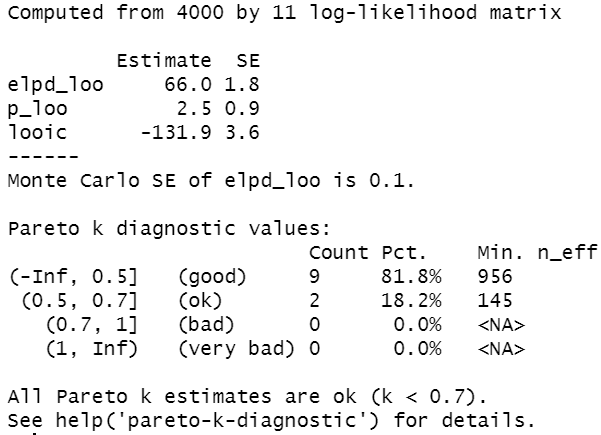


**Supplementary Figure 2. Diagnostics of emulator’s predictive accuracy for modeling hypertension prevalence**


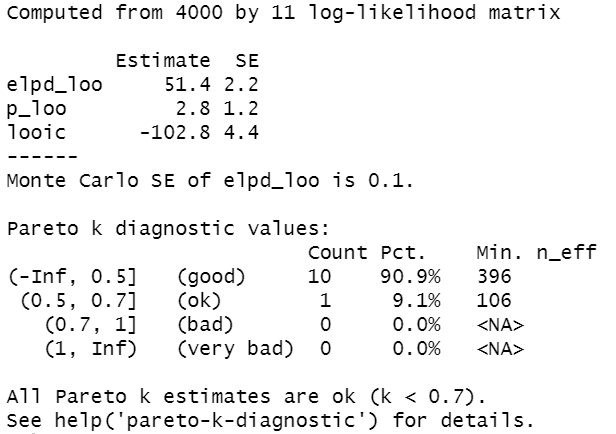


**Supplementary Figure 3. Diagnostics of emulator’s predictive accuracy for modeling HIV and hypertension comorbidity**


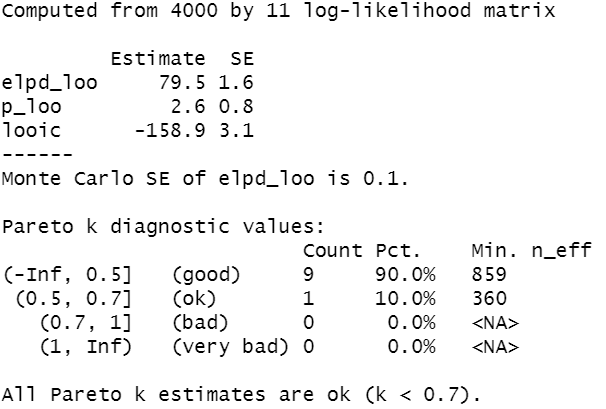


**Supplementary Figure 4. Diagnostics of emulator’s predictive accuracy for modeling depression among people living with HIV**


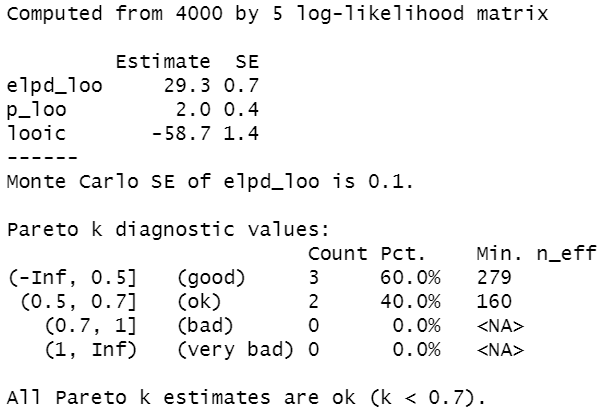


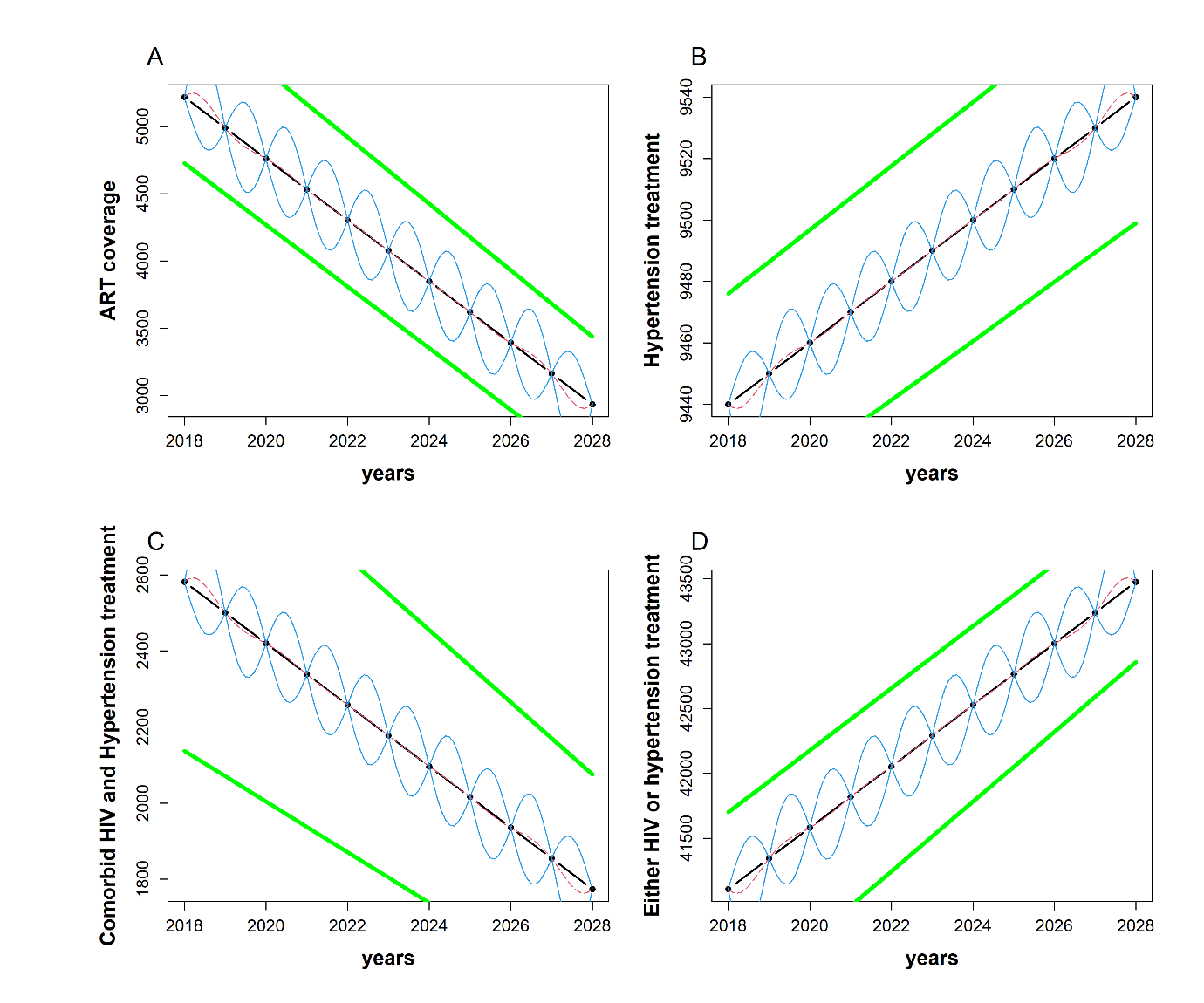


**Supplementary Figure 5: Gaussian process emulator of disease burden when (a) ART coverage targets are met, (b) Hypertension treatment targets are met, (c) Comorbid HIV & Hypertension treatment targets are met, and (d) Either HIV or hypertension treatment targets are met.** Considering the inMODELA simulator for Kenya^29^, the ART coverage target assumed 90% of people with HIV were aware of their status and 90% of those people were enrolled in HIV care; the hypertension treatment target assumed 50% of confirmed hypertensive patients were receiving drug therapy and counselling. At each year, black dots represent the selected design points used to fit the emulator, the blue curved lines represent the predicted 95% uncertainty intervals for each design point, the dashed red lines are the mean prevalence for a given year plotted as a function presenting the emulator’s predictions, and the green thick lines outside are the 95% confidence intervals for the original simulators.


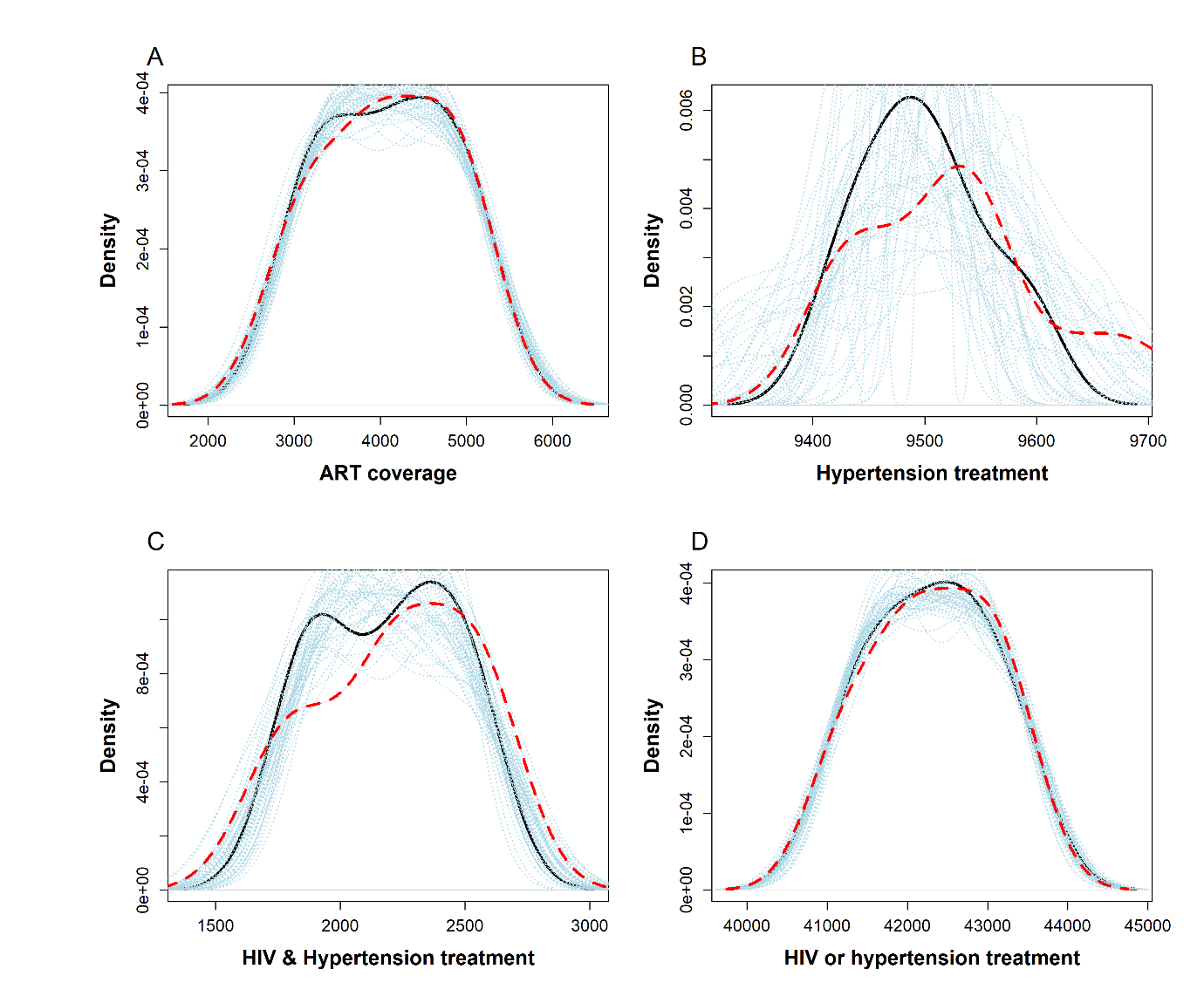


**Supplementary Figure 6:** **Gaussian posterior predictive distributions for (a) achieved ART coverage targets, (b) achieved Hypertension treatment targets, (c) achieved comorbid HIV & Hypertension treatment targets, and (d) achieved either HIV or hypertension treatment targets.** Considering the inMODELA simulator for Kenya^29^, the ART coverage target assumed 90% of people with HIV were aware of their status and 90% of those people were enrolled in HIV care; the hypertension treatment target assumed 50% of confirmed hypertensive patients were receiving drug therapy and counselling. The solid black lines represent the distribution of *y*; the dashed red lines represent the *y* posterior predictive distribution and the dotted light-blue lines represent the distribution of simulations. The density values of the plots are different as generated by the system due to the differences in disease-specific prevalence values on the x-axis.

**Supplementary Figure 7. Diagnostics of emulator’s predictive accuracy for modeling HIV prevalence, assuming Kenya’s ART treatment targets are achieved (i.e., 90% of people with HIV are aware of their status and 90% of those people are enrolled in HIV care)**


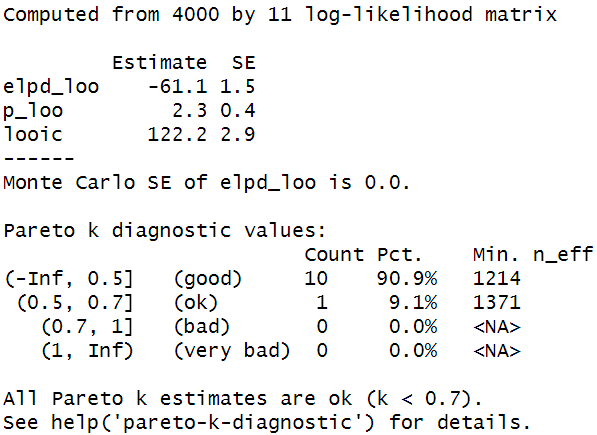


**Supplementary Figure 8. Diagnostics of emulator’s predictive accuracy for modeling hypertension prevalence, assuming Kenya’s hypertension treatment targets are achieved (i.e., 50% of confirmed hypertensive patients are receiving drug therapy and counselling.)**


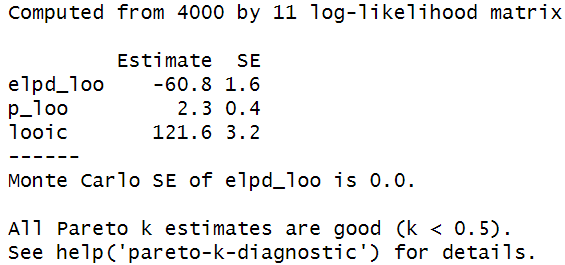


**Supplementary Figure 9. Diagnostics of emulator’s predictive accuracy for modeling comorbid HIV and hypertension prevalence, assuming Kenya’s ART coverage targets and hypertension treatment targets are achieved (i.e., 90% of people with HIV are aware of their status and 90% of those people are enrolled in HIV care, and 50% of confirmed hypertensive patients are receiving drug therapy and counselling)**


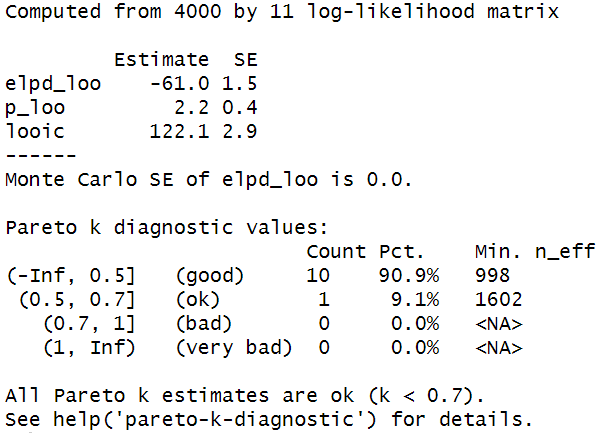


**Supplementary Figure 10. Diagnostics of emulator’s predictive accuracy for modeling comorbid HIV and hypertension prevalence, assuming Kenya’s ART coverage targets or hypertension treatment targets are achieved (i.e., 90% of people with HIV are aware of their status and 90% of those people are enrolled in HIV care, or 50% of confirmed hypertensive patients are receiving drug therapy and counselling)**


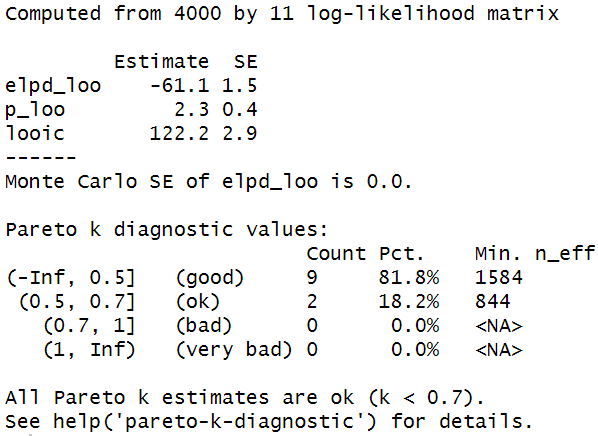

Supplement: Supplementary file 1 — Additional file 1. [file 12874_2024_2149_MOESM1_ESM.docx]
